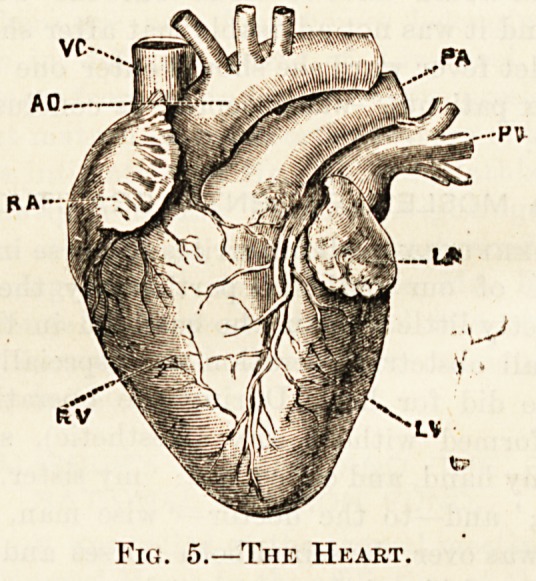# "The Hospital" Nursing Mirror

**Published:** 1900-04-14

**Authors:** 


### "The Hospital" Nursing Mirror.

Being the Nursing Section of "The Hospital."

[Contributions for this Section of "The Hospital" should be addressed to the Editor, The Hospital, 28 & 29, Southampton Street, Strand, London, W.C., and should have the word "Nursing" plainly written in left-hand top corner of the envelope.]

### Notes on News from the Nursing World.

### THE "PRINCESS OF WALES" HOSPITAL SHIP.

Our excellent contemporary, *Lloyd's Newspaper*, seems disposed to take up the position that the testimony of a single individual is worth more than that of a considerable number; but in attempting to extenuate the minor complaints of Sergeant Harper about his treatment on the "Princess of Wales" hospital ship, it ignores the major charges. By far the most serious of those attributed to Sergeant Harper were, to use his own words, "since we left Table Bay I haven't known what a square meal is," and "to speak the truth, I heard more complaints on that one voyage in the 'Princess of Wales' than I heard during seven years I spent in the army." We did not think it worth while to notice such a grievance as that "to get salt we had to wait half-an-hour." But in the interview of our Commissioner with several of the patients at the Princess of Wales's Hospital for the Wounded at Surbiton, he was able to obtain the most complete and emphatic refutation of the graver assertions which our contemporary now so strangely puts aside. The only other point of any importance is, that *Lloyd's* cites the fact that supper is given at seven at "The Gables" as a reason why it should also be given on the "Princess of Wales" hospital ship. The difference, however, is, that while all hospital ships are managed on precisely the same principles as Netley, Woolwich, and other military hospitals, a little latitude in the matter of meals is naturally allowed at Surbiton, where Mr. and Mrs. Cooper provide everything entirely at their own expense. If the War Office could see their way to add a light supper in all the military hospitals and ships, the extra meal would, we believe, be much appreciated, but this is a matter which applies to military hospitals in general, and not merely to one particular hospital ship.

### A NEW NURSING HOME FOR DUBLIN.

On Monday Princess Christian laid the foundation stone of the new nursing home attached to the City of Dublin Hospital, of which the Queen is patroness. There will be sleeping accommodation for 30 nurses, each having a separate cubicle with its own window. A recreation-room, opening on to a lawn, and a large dining-room, will also be provided; and there will be a bedroom, sitting-room, and offices for the matron. An address of welcome to the Princess was read, in which the hope was expressed that the home would be built and in working order at no distant date. Her Royal Highness, in her reply, said that "she had always taken the greatest interest in everything connected with hospital nursing, and that she knew, from many years' experience, the numerous benefits conferred on the suffering poor by such institutions." She subsequently, amid loud cheers from a large company, intimated that she had been commanded by her mother, the Queen, to say that the hospital should henceforth be known as the Royal City of Dublin Hospital.

### SIXTEEN HOURS A DAY AT LADYSMITH.

"One of the Natal Volunteers" writes us:—"All of us, including the doctors, have nothing but praise for the nurses. At Intombi Camp all the nursing had to be done in tents, the nurses having to pass in and out from tent to tent in the burning sun, or the pouring rain, thus exposing themselves to every change of temperature. Many of them were burnt and tanned beyond recognition, but on the whole, considering the terrifically hard work, the health of the staff was wonderful. When some were ill, their places had to be filled by the others, so that it was no unusual event for the nurses to work sixteen hours a day. Everyone was cheerful and bright, however, notwithstanding the mud through which they all often had to wade. The chief cause of distress to them was the absence of suitable food to give to their invalids. Starch puddings pulled many a poor fellow through enteric, but when the stock was consumed, they had to fall back upon violet powder. Sweetened lozenges not required by the doctors were eagerly sought after. Horse-soup was found more palatable when called 'chevreuil,' and was really fairly good."

### A DINNER TO A NURSE.

The same correspondent says, "Those nurses who remained in the town were under shell fire all the time, and the nurse in charge of the town hospital unfortunately contracted enteric. Her position was an onerous one, as during the night, the doctors sleeping 'out,' everything devolved upon her. At the end of seven weeks she was about again, and the first time she went out she was deeply touched by the number of men and women, quite unknown to her, who came and expressed their joy to see her better. Some of the volunteers gave a dinner to celebrate the fact of her recovery, at which she was the honoured guest. The menu consisted of chevreuil soup, haunch of roast horse, and a handful of spinach, which had cost the hosts 15s. But no one grudged the money, the men were so proud to have procured it."

### THE NURSES OF WEST AUSTRALIA.

A number of cuttings of letters contributed to West Australian papers respecting the nurses in that Colony have reached us. It appears from these to be a matter of complaint that the members of the profession who were accepted for service in South Africa have not been treated as they expected. One reported grievance is the delay between the appointment of the nurses and their departure to the seat of war, and a satirical contributor, dating from Perth, suggests that Lord Roberts should be invited to suspend the campaign for three months, "so as to allow the West Australian nurses to get a start on their journey." Another grievance is, that the local committee intend to send the nurses as steerage passengers. We cannot believe, however, that better accommodation will not be provided for them. These, and other points about nursing in Western Australia, and especially the alleged opening for nurses from England in some portions of the Colony, can be dealt with more authoritatively in a subsequent issue, when the Governor, who is now on his way to this country, reaches London.

### THE CONCERT FOR THE "MAINE."

THE centre of attraction at the concert given at the Crystal Palace last week, in aid of the hospital Ship "Maine," by the pupils of the Royal Normal College for the Blind, were the items on the programme rendered by the blind pupils themselves. Amongst these were a charming Polonaise for piano and orchestra, the solo part of which was played on the pianoforte by the composer, Master Pegg, a boy of about fifteen, who is a pupil of the Royal Normal College; and Hertz's "Duo du Couronnement," played by Miss Mabel Davis and Miss Emily Lucas. The singing of the choir, too, was exceedingly good and spirited, and reflected great credit on the training and teaching enjoyed by these blind pupils. Madame Albani very kindly contributed three songs in her usual delightful manner, and Mr. Watkin Mills and others also added to the enjoyment of the afternoon.

### PAY UP, LEICESTER!

IT is not pleasant to learn from the report of the Leicester Institution for Trained Nurses that "the finances of the branch remain in their usual unsatisfactory condition." And yet all that is wanted "to place the work on a sound financial footing" is the addition of £100 to the annual income, while it seems that another hundred would be required in order to make the scale of the operations adequate to the ever-growing wants of the town. Considering that Leicester is one of the most prosperous, as well as one of the most go-ahead, towns in the United Kingdom, it seems incredible that there should be any difficulty about raising even £200 a year. But half that sum ought to be readily obtainable. The *Leicester Daily Post*, says "Only those who have been behind the scenes, and have thus obtained a flood of light on the work that is being done in the eleven districts into which this borough is divided, can have even a faint conception of the real magnitude of the service that is thus rendered to the sick and suffering poor. In ordinary private nursing also, the institution still fulfils its important mission." In the face of such testimony as this, we shall be exceedingly surprised and disappointed if the treasurer should again be driven to the desperate expedient of meeting current expenses by a draft on a reserve fund which, instead of being allocated to such a purpose ought to be substantially increased.

### THE MATRON OF ST. GEORGE'S HOSPITAL, FULHAM ROAD.

MR. EVERITT, one of the guardians of St. George's, Hanover Square, having refused to withdraw the charges of extravagance against Miss Hampson, matron of the infirmary, the latter has very properly decided to bring the matter before the Local Government Board. No doubt a speaker like Mr. Everitt is not to be taken altogether seriously. In the speech in which he persisted in his allegations, Mr. Everitt said that "all women are extravagant." This is quite sufficient to discredit the opinion he has expressed about Miss Hampson. At the same time, it is quite intolerable that personal charges should be recklessly made against officials, and then excused by such trivial generalities, and we hope that the Local Government Board will lose no time in dealing with the question. But in any case, the tendency shown by many of the guardians to resist all measures of reform would seem to render it desirable that an enquiry should be made into the entire administration of affairs by the Guardians of St. George's Union.

### PENSIONS FOR LIVERPOOL DISTRICT NURSES.

AT the annual meeting of the Queen Victoria Liverpool District Nursing Association, Mr. John Henderson, recognising the desirability of establishing a superannuation fund for the benefit of the nurses, offered to contribute £500 for the purpose of starting it. The offer was, of course, thankfully accepted, and it was stated that the handsome donation would enable the committee to contribute to the Royal National Pension Fund without, perhaps, forming a local fund, and also to make provision in cases of incapacity and illness. There are now four homes belonging to the Association in different parts of Liverpool, each of which contains from six to twelve nurses, presided over by a district matron. A noteworthy feature of the meeting was the intimation of Mr. William Rathbone, that he had recently seen Miss Florence Nightingale, who, he said, was still able to retain the most vivid interest in the nursing work. According to Mr. Rathbone, Miss Nightingale's home at Liverpool is a stronghold of district nursing, and she is very anxious that it should keep in the front.

### THE SALISBURY NURSES AND THE ROYAL NATIONAL PENSION FUND.

ATTENTION was called at the annual meeting of the Salisbury Nurses' Home to the affiliation of the institution with the Royal National Benevolent Association for Nurses, under which scheme the former last year paid a contribution of £174 17s. 1d. Mr. Pinckney hoped that supporters of the Home would note that fact and continue their subscriptions until the institution was out of the wood, because there were old nurses who had to be pensioned and taken care of. Fifteen of the nurses had joined the scheme to secure the annuity of £15 at the age of 50. The staff of the institution numbers five less than it did in the previous year, a circumstance which is due rather to lack of financial support than to diminished number of cases of sickness.

### THE BRISTOL DISTRICT NURSES.

AT the annual meeting of the Bristol District Society several speakers alluded in terms of warm commendation to the admirable work done by the nurses. Dr. Parker said it was greatly appreciated by the poor, and "it would be a calamity to any district if it were to lose its nurse." This being so, it is not satisfactory, so far as the public are concerned, to learn from the report that out of seventeen districts, only three are fully paid for. The committee do not like the idea of withdrawing nurses from those districts which fall very short of the payments expected from them, but unless there is a rally on the part of subscribers, it may become absolutely necessary for them to adopt that unwelcome course. It is a matter of some surprise that some of the Nonconformist churches refrain from giving any support to the District Nurses Fund, although a considerable proportion of the patients attended by the nurses are Nonconformists, and the society itself knows no religious distinctions.

### THE NURSING STAFF AT THE EXETER SANATORIUM.

At a recent meeting of the Exeter City Council it was reported that the matron of the Devon and Exeter Hospital recommended the appointment of three staff nurses at a salary of £26 a year, and four probationers, and this was adopted. Hitherto, the nurses at the sanatorium have been supplied by the hospital authorities, but under the new arrangement the committee of the Council will teach their own staff at the sanatorium, and we gather that they consider it will save expense. A curious effort was made at the meeting to prevent the appointment of two wardmaids instead of one, but the councillors who advocated this policy were very much in a minority. The argument that "one wardmaid would have to scrub out the whole of the wards, and it was not advisable that after she had been in a scarlet fever ward she should enter one containing small-pox patients," was accepted as conclusive by the majority.

### A MOSLEM PATIENT'S GRATITUDE.

Wonderfully grateful, writes a nurse in the East, are some of our patients, particularly the Moslems. "One pretty little woman who has been in the hospital for a small obstetric operation was especially thankful for all we did for her. During the operation (which was performed without any anæsthetic), she caught hold of my hand, and called out: 'my sister, my sister, help me;' and—to the doctor—'wise man, help me!' After it was over she seized both nurses and doctor by the hand, and kissed them over and over again. Later on she drew from her nose a precious ornament—a piece of coral—which she begged me to accept, at the same time she told the doctor to bore a hole for it in my nose! But the final piece of generosity was to offer me a charm she wore next to her skin. It looked like a little leather purse sewn up. 'Inside,' she said, 'is writing from a Carmelite Monk,' and she told me further, that among other things, it had power to give or withhold sleep! Knowing what such a sacrifice would cost the poor soul, I declined her gift, but must own I should have much liked to possess her cure for insomnia—even if only as a curio!"

### TOO STRONG FOR THE FURNITURE.

An English matron, with American experience, sends us the following account of an unusual accident: "Towards the close of a summer's evening I was busy with the battery and massage at a paralysis case half way down a long ward of 24 beds. The bath-room, with its large, deep bath of highly polished copper, was at the far end of the ward, and just now occupied by Mrs. D——, a patient of 20 stone, and a convalescent, for the hospital rules forbade any patient to take a bath alone. Mrs. D—— had become an in-patient because of her obesity. Hot-air baths, banting, &c., all failed of their purpose; her flesh obstinately remained 'too, too solid.' Suddenly the whole ward was startled by the most piercing screams and shrieks from the bath-room. Nurse —— and I rushed in, to find ourselves enveloped in steam; the adipose patient lying in the bath, with boiling water from the pipe pouring on to her. To get her unwieldly person into the bath, she had taken hold of the large hot-water tap, similar to those in the P. and O. steamer bath-rooms, and, not constructed for the strain of 20 stone, it had separated from the pipe, throwing her into the bath, and releasing the boiling water. Nurse —— seized hold of the large draw-sheet, which it was customary to keep in the bathroom, and stuffed it into the pipe, holding it there with imminent risk of scalded hands. It was impossible for us by ourselves to get the poor woman out of the bath; fortunately one of the house surgeons, attracted by the screams, came to the rescue, and with the help of a blanket (the supply of water having in the meanwhile been quickly cut off) we managed to get the poor creature from bathroom to bed, where dressings awaited her. The whole of her back and both arms were very badly scalded. She was in bed for six weeks, and suffered terrible pain. The remedy, though severe, effected a marked change in her weight, and she left the hospital a thinner, if a sadder, woman. I cannot forbear to point a moral. That very morning I had found the big draw-sheet, mentioned above as doing such signal service, whose rightful place was the bath-room, left by some untidy nurse in the lavatory on the opposite side of the ward; though not the delinquent, I took it back to the bathroom. What we should have done without it at the time of the accident I cannot imagine, for there was nothing else at hand to stop the torrent of boiling water."

### SHORT ITEMS.

The Duke of Portland has been elected chairman of the Queen's Commemoration Fund, on behalf of the Queen's Jubilee Institute for Nurses, in succession to the late Duke of Westminster.—In the annual report of "Fridenheim," the excellently-managed Home of Peace for the Dying, at Swiss Cottage, Miss Davidson, the honorary superintendent, expresses her regret that there is nothing further to announce with regard to the long-talked-of Nurses' Home, and her hope that ere many months are over it may be nearer accomplishment than seems probable at present.—The annual meeting of the Children's Fresh-Air Mission will be held on Thursday, the 26th instant, at Holborn Town Hall. The report for 1899 shows that 3,171 poor and ailing children were sent into the country last year for change of air.—A warm tribute to the excellence of the management of the Infectious Diseases Hospital at Plymouth has been paid by Mr. J. H. Worman, the United States Consul at Munich. This gentleman and his family were passengers by one of the steamers calling at Plymouth, and a child of his was removed to the hospital, suffering from diphtheria. Mr. Worman, in a letter to the Sanitary Committee, says he cannot acknowledge too strongly "the exceeding great care exercised by the matron and her assistants, as well as the great watchfulness of your distinguished medical adviser.'

### Lectures on Nursing for Probationers.

By E. MacDowel Cosgrave, M.D., &c., Lecturer to the Dublin Metropolitan Technical School for Nurses.

### III.—THE THORAX.

The thorax in the skeleton is bounded in front by the breastbone or sternum, the flat bone to which the collar-bones and ribs are attached, and which ends below in the ensiform cartilage. At the back are the 12 dorsal vertebræ and part of the 12 ribs which also form the lateral boundaries, the cartilages joining the sternum in front. The upper or true ribs are joined to the sternum by their cartilages; of the remaining five false ribs three are attached by their cartilages *indirectly* to the sternum, through the cartilages of the seventh rib. Muscles fill up the remaining spaces. Thus, running from the lower part of one rib to the upper part of the rib below are two sheets of muscle, the external intercostals run downwards and forwards, the internal intercostals run downwards and backwards; the space between contains connective tissue in which run the blood-vessels and nerves, safely placed close under the lower edge of the upper rib. This arrangement is found in all the interspaces of the ribs.

The floor of the abdomen is closed in by a large sheet of muscle called the diaphragm, this is attached by its tendons to the ensiform cartilage at the lower end of the sternum, to the internal surface of the six lower ribs, and from the bodies of some of the lumbar vertebræ by its "pillars." It is arched; on it rest the lungs. When air is drawn in in breathing, the external intercostal muscles raise the ribs, and the diaphragm flattens, thus the cavity of the chest is increased from front to back and from above downwards.

The chest contains the lungs and heart. The lungs are spongy, elastic organs; a little piece cut off and thrown into water floats because it contains a quantity of air in its cells. Each lung is contained in a double bag of smooth shining material—the pleura—one layer of which covers the lungs, the other lining the inside of the chest cavity; the surfaces are moist, and so allow movement without friction.

The lungs are only attached by their "roots" just over the heart, each root contains a bronchus to convey air, a pulmonary artery and two pulmonary veins, and the bronchial vessels supplying blood to the lung tissues; there are also lymphatic vessels and glands, and, of course, connective tissue. The roots attach the rest of the lungs to the trachea and the great vessels of the heart, the rest of the lungs lie free in the pleural cavity. The right lung is the largest, and is divided into three lobes; the left is the longest and has two lobes. Air passes down the trachea or windpipe and then goes into the right or left bronchus; these split into bronchial tubes which get smaller and smaller, finally opening into dilated air sacs or cells, through the walls of which the impure blood gets rid of its carbonic acid gas and takes in oxygen. The walls of the trachea, bronchi, and bronchial tubes have curved plates of cartilage to keep them open; the walls of the air-cells contain muscular fibres, and so the cells expand and contract during respiration.

Most of the chest gives a hollow, resonant note when struck, but there is one dull spot about the size of a shilling to the front of the left side. This is caused by the apex of the heart which here comes to the surface.

The heart is placed in a slanting direction behind the lower two-thirds of the sternum, its base is highest up and furthest back, its apex points forward and towards the left; at each beat of the heart the contraction of its muscular walls causes the apex to strike the chest-wall; this can be seen or felt two inches lower than the left nipple, and an inch closer to the middle line; this is just below the fifth rib, where it joins its cartilage. The heart weighs about three quarters of a pound, and is a little smaller than the closed hand; if the right fist be placed across the sternum, the wrist being kept back and the knuckle of the first finger projecting downwards to the left, it will give a good idea of the position of the heart, the wrist representing the base and the knuckle the apex. The heart is contained in a closed double bag—the pericardium. This has a smooth surface, like the pleura, and contains a little fluid, enabling the movements of the heart to take place without friction. The heart is free in this bag, being attached at the base where the large vessels are given off; this is at the level of the roots of the lungs. The heart is built up of two halves, right and left, the right being most in front. In heart disease either or both sides may be enlarged, and then the dulness covers a larger space, spreading either to the right or left or to both.

The chest also contains the œsophagus or gullet which runs down the front of the spine, passing out through an opening in the diaphragm. The aorta, or chief blood-vessel, rises up

Fig. 5.—The Heart.

from the heart, and arching over it runs down the spine behind the œsophagus, passing into the abdomen through another opening in the diaphragm.

The chest may be altered in shape in many ways. It may be: (1) Pigeon-breasted, when the sternum is pushed forward, the ribs being flattened so that a section of the chest is more triangular than oval. This follows rickets, the unnaturally soft ribs having been drawn in during inspiration. (2) Alar, the shoulder-blades projecting outwards like wings. This occurs in delicate people, with badly-developed chests and weak muscles. (3) Barrel-shaped, as occurs in some diseases with obstruction to expiration; the section of the chest is more round than oval, and the upper part of the chest hardly moves in respiration. (4) As a result of tight lacing the lower ribs may be drawn in and even overlap. (5) Flattening of the upper part is seen in early cases of consumption, the clavicles being unduly prominent. (6) In pleurisy with effusion the affected side may be larger; when the effusion is absorbed the side may contract from the lung being tied down by adhesions and unable to expand.

A few examples of conditions requiring surgical treatment may be given:—

Fracture of Collar-Bone.—Here, owing to the loss of support of the collar-bone, the shoulder drops and is drawn towards the middle line. The treatment is to place a firm pad in the axilla or armpit, and to bandage the elbow tightly to the side, the hand being also fixed and supported by a sling.

Fracture of Rib.—A tight band or strips of plaster are wound round the body at the level of the injury; this prevents the injured part being moved during respiration.

TRACHEOTOMY has sometimes to be performed to relieve obstruction of breathing, the surgeon cutting into the trachea a little above the top of the sternum, and introducing a tube which curves downwards, and through which breathing takes place.

TAPPING may be necessary in pleuritic effusion. The place selected is towards the back, at the level of the lower angle of the scapula, so as to reach the lower part of the cavity. A hollow needle is pushed in through the space between two ribs, keeping close to the lower one so as to avoid the vessels.

When there is empyema—pus in the pleura—a larger opening is made, and the cavity may require to be washed out.

### East-End Mother's Home.

THE annual meeting of the governors and friends of the East-End Mothers' Home was held last Friday afternoon. The Rev. W. MUIRHEAD presided. The meeting was well attended. Several subscribers who had not previously seen the improvements took this opportunity of doing so, and afterwards expressed their pleasure and satisfaction to the matron. Mr. Lacy, the secretary, read the report of the committee of management. The number of in-patients for the year was 264. The average number of beds occupied was twelve. Fourteen midwifery pupils out of seventeen obtained the L.O.S., and twenty-two maternity nurses were trained. During the year the debt against the charity has been reduced by £100, and a very urgent appeal is made for further support. The home has lost two friends through death—the Hon. Mrs. Stuart Wortley, one of the founders of the charity, and Mr. Carter, a former chairman. The medical report showed that of 264 in-patients, 255 children were born alive. Amongst the 255 out-patients, 251 children were born alive. The extra accommodation has proved most useful, enabling the authorities to retain cases requiring care until fully convalescent. The matron's report showed considerable changes in the nursing staff; two members have entered general hospitals to train for three-year certificates, and a third has taken up private work.

### Appointments.

GORE FARM CONVALESCENT FEVER AND SMALL-POX HOSPITAL, DARENTH.—Miss Jessie Rebecca Warr has been appointed Assistant Matron. She was trained at the Evelina Hospital and King's College Hospital. Her subsequent appointments have been those of charge nurse at the South-Western Hospital, ward sister at Monsall Fever Hospital, and night superintendent at Belfast Royal Hospital.

PARK FEVER HOSPITAL, LEWISHAM.—Miss Ellen Buxton has been appointed Matron. She was trained at St. John's House, Charing Cross Hospital, and the Metropolitan Hospital. Subsequently she has been charge nurse, superintendent of nurses, and assistant matron at the North-Eastern Hospital, Tottenham, and matron of the Isolation Hospital, Norwich.

GROVE FEVER HOSPITAL, LOWER TOOTING.—Miss Elizabeth Jane West has been appointed Matron. She was trained at St. Bartholomew's Hospital, and has since been staff nurse at the same institution, night superintendent at Chelsea Infirmary, and assistant matron at the Fountain Hospital, Tooting.

### Co-operation Among Older Nurses.

SINCE the foundation of the first co-operation of nurses under the auspices of Sir Henry Burdett in 1891 a great deal has been done to rescue the nurses' calling from the tender mercies of the "middle man," who used unimpeded to absorb the major part of their earnings. The recently trained nurse now finds no difficulty in getting a place on the staff of institutions modelled on the lines of the original "Nurses Co-op.," in which a deduction of 7½ per cent. from her earnings proves sufficient for all office expenses and often leaves a small margin to accumulate.

The employment, however, of older nurses, women of 40 and upwards, is still completely without organisation, and few people except the nurses themselves are aware of the extent to which these women are taken advantage of. Only a short time back it was the invariable custom of doctors in general practice to have their own staff of nurses and to send for them direct to their home address as they required them. But of late years doctors have cared less and less to undertake the troublesome office of keeping themselves acquainted with the engagements of their nurses, and they now commonly refer to some institute which can be trusted to supply their needs. Many older nurses have fallen in consequence out of continuous employment, for "not over forty" is the invariable answer to any applicant desirous of joining a good co-operation. There are many small nursing institutes and homes which will take older women, but the percentage demanded on their earnings is never less than 3s. 6d. in the guinea, or 17½ per cent., and often amounts to 20, or even 25 per cent. (7s. in each guinea). Many women who have had excellent training, and served diligently, find all the advantages of their former good work slip from them as time goes on in the heart-sick waiting between cases. And this is the more deeply to be regretted because there is an undoubted desire on the part of the medical profession and the public alike to avail themselves of the services of experienced middle-aged women if they can be sure that their character is good and that they have a sound knowledge of their business. Indeed there are many reasons, both social and medical, which may render the offices of an older woman in illness indispensable. We are glad to learn that an effort is being made by the Royal British Nurses' Association to supply this long felt want in the nursing world by founding a co-operation of older, or, as it is proposed to call them, "auxiliary" nurses. The staff is to be made up entirely of nurses between the ages of forty and sixty who have had at least three years' hospital experience, and whose nursing career has been unimpeachable. The percentage on their earnings will be 7½ per cent. The association, from the fact of its presenting a combined front of doctors and nurses, is peculiarly well equipped for an undertaking of this sort. The difficulties in the way of combining middle-aged women for their own advantage are likely to prove considerable, but we cannot believe that they are insuperable, and we wish the new enterprise every success. Nurses desirous of joining the new staff, who are not already members of the Royal British Nurses' Association, should apply in the first instance for forms of registration to the secretary, 17, Old Cavendish Street, W.

### OUR CONVALESCENT FUND.

WE acknowledge the receipt of 2s. 6d. from Miss Dinwoodie, and 2s. 6d. from Nurse H. M. Walker, to our Convalescent Fund. The holiday season is fast approaching, and we should be glad if our subscribers will send in their contributions.

### How the Dublin Nurses Saw the Queen.

By a Special Correspondent.

The sun shone clear and cold, the north-west wind blew out a forest of flags against the stone-blue sky. Merrion Square was wreathed and chained with flowers; from almost every window scarlet, blue, green, and daffodil draperies hung out, and the tall crimson poles supporting the balconies mingled with the long vista of Venetian masts in the street, in strange mimicry of a scarlet fleet at anchor.

The crowd was not of the densest, for the long ten miles of the route gave ample accommodation to all spectators. But the glittering lines of Lancers guarding the kerbstone had quite enough to do to keep back the people from spreading over the roadway; and when their horses turned restive, as they did now and then, it looked as if there was going to be work for the ambulance waggonette, which fortunately, however, remained idle all the morning. Flying its Red Cross flag conspicuously, it stood at the corner of the square, tenanted by a couple of cool-eyed, smart young soldiers, in all probability better trained in accident work than many of the nurses who glanced at them with professional interest and curiosity from time to time.

### A Probationer's Complaint.

"I am sure I wish we had more chance of perfecting ourselves in accident dressings," complained the nurse in blue at the corner of the kerb to the one in red on the outside car. "Once you put on a bonnet and cloak, you are expected to know everything. The public think you a mischievous idiot if you can't find and compress a brachial artery through a coat and shirt with your gloves on, kneeling in the mud with an excited crowd shoving you about, or diagnose apoplexy from drunkenness, first shot; or reduce a compound comminuted fracture of the femur with your pocket-handkerchief and two sticks of barley sugar."

"Now you're exaggerating," said the nurse in red, severely.

"Hardly a bit. What chance does a pro. in an average hospital get of treating accidents in a sensible, first-aid way? Holding the corrosive basin and handing bandages doesn't teach you much. Of course, it isn't supposed to be your business, but the public make it so."

"I shouldn't be afraid of 'first-aiding' anything," said the nurse in red.

"Because you have been eight years qualified, and seen and done everything, as one does in time. But the opportunities are so scrappy and seldom that a nurse who has just got her badge is very little good if you knock her off the regular tram-line of ward work."

"All very true, but I don't want to talk shop now," yawned the nurse in red. What a lot of cloaks there are!"

### Nurses in Force.

There were, indeed, for Merrion Square is within easy distance of six big hospitals, and rules had everywhere been strained to breaking point to allow of the greatest possible number being present at the Royal entry. Lucky, indeed, on Wednesday were the nurses of the great Roman Catholic hospitals, for the good sisters of mercy and charity, who never leave the hospital walls except in case of very special business, undertook the whole day's work themselves, and allowed any nurse who wished to take holiday. For once the dignified black-habited mistresses of each ward went about dusting, serving meals, dressing, washing up, drawing and making beds, with the energy of three "pro's" apiece, and on the return to headquarters of the nurses with the blue cloaks and veils, the nurses with the blue cloaks and big bows, the nurses with the purple cloaks and velvet bonnets, they were laughingly informed that the patients had never been as well cared for as on the day of the Royal entry.

### How the Nurses were Divided.

In the hospitals carried on by secular authorities, Sunday hours were observed and the day divided into halves. One half of the nurses went to see the procession of the morning, the other half to see the illuminations at night. Those on night duty had the procession or the illuminations, according to the custom of the hospitals. Where the night nurses went to bed on coming off duty the evening was given, while in those hospitals where bedtime is about noon the hour was stretched to permit a view of the procession; but after the long night's vigil, and the tiring rush of the early morning work, the eyes that looked out on the sea of shifting colour were sometimes very heavy. Then the bonneted head would nod downwards towards the rail of the stand, and the tired night nurse would start awake with a feeling of horrible guilt—to realise that she had not, after all, dozed off for a fatal "one minute" by the ward fire, but that the sun was shining and the free wind was blowing, and she must keep her wits about her, for the Queen was surely due.

### Thh Queen Passes.

From the direction of Leeson Street a dim, thunderous murmur began to rise, swelling into a far-away roar. The Queen had reached the "City Gate"—the quaint old ceremony of admission was being gone through. A pause of quiet, and then the far-off roar commenced again, gathering and swelling down the street like an Atlantic roller sweeping in upon the wild West coast. The crowd began to heave and surge; the Lancers backed their clattering horses; the first three carriages of assorted Royalties trotted after a gorgeous body of cavalry, little noticed or greeted, for every eye was strained towards the fourth carriage. It came, neared, passed, and disappeared in the distance, and the crowd, which had now broken all bounds, pressed cheering round the Royal equipage. The old, kindly, motherly, grave, noble face, heavily veiled and spectacled, the feeble, aged frame bowing slightly still to right and left after the long seven miles of salutations, these passed away in a second; yet they were what the cheering thousands had come out to see; what none of the present generation of Irish, save those who make visits to the sister island, had ever in their lives had the opportunity of viewing.

### Nurses' Politics.

There are all shades of politics to be found among Dublin nurses; for, busy as their lives are, they cannot escape the strong political currents of the country. Many of the cloaked and bonneted figures had worn shamrocks tied with crape on St. Patrick's Day; others now displayed Transvaal buttons on their capes; others, again, showed green ribbons, or even tricolour and green, among the numberless badges of Union Jacks and roses. But this had apparently no effect upon the nurses' personal welcome to her Majesty, for Nationalists and Unionists alike waved their handkerchiefs and cheered with Celtic fervour, their long veils flying in the cold, bright wind, and their snowy cuffs flashing up and down under the flapping capes as they enthusiastically did their share towards welcoming the Queen.

### "General Bobs."

At night, the nurses turned out in full force to see the illuminations, between eight and ten. One little general of a matron (occasionally nicknamed "Bobs," from a very perceptible parallelism of character) led a charge of more than twenty "pro.'s" through the "thickest of the fight" in Grafton Street, each nurse holding tightly to her immediate precursor, and the indomitable general leading the way like Henry of Navarre. Most of the hospitals contributed their own share of coloured lamps to the festivities, though none were very brilliantly adorned. The chimes of eleven o'clock saw matrons, sisters, staffs, and "pro.'s" finally at rest the city through, after a day which was agreed by all to be a Royal red-letter one, in spite of its producing more fatigue than any day in the wards.

### The Nurses of the Imperial Yeomanry Hospital.

By One of Them.

Writing from Deelfontein under date of March 18th, a correspondent says: On Wednesday last the "Guelph" anchored in Table Bay at four a.m. Later we went ashore and occupied several pleasant hours buying little necessaries and looking round. We spent that night on board and all next day, arriving in the docks at six p.m., where we were met, and speedily our luggage was landed. As we filed across the gangway the "Tommies" heartily cheered us again and again, bringing a lump to our throats as we thought of the perfect safety to which we were going as compared with their uncertain prospects. We were entrained at the goods station, from which the troops always start. We occupied two corridor carriages at the end of a luggage train, leaving at ten p.m. We were very comfortable, as we were able to have berths fixed up at night, which we thoroughly appreciated.

### The Journey by Land.

We had an intensely interesting journey along the whole 476 miles. When we awoke on Friday morning it was to find ourselves rapidly approaching the Herx River Mountains, and for the five hours that our train wound its way round and about and along the sides of the mountains (often doubling back and running parallel for quite a long distance, with a portion of the line we had quitted just on the other side of a deep and comparatively narrow valley) we had a most glorious outlook. It brought vividly to our minds the episode of Spion Kop and all the difficulties our brave men had to contend with there, for here we saw many kopjes rising from amidst the mountain range, which we imagined must resemble Spion Kop. Land being cheap and labour expensive is, I suppose, the explanation of the fact that there are only two short tunnels the entire distance. We came at intervals upon pickets of "gentlemen in khaki" guarding the line of communication, being particularly strong in places where there were bridges or watering stations. As we slowed down there would be a rush from the camp, and soon we would be surrounded, and chatting animatedly, till we steamed slowly away, when, without exception, Tommy cheered, and cheered, and cheered again. It was a right royal reception we got from them all. The great excitement was the concentration of the rebels, who were often within a few miles of the railway—in one place their signals were noticed, beginning with one fire till a dozen or more were observed flashing in different directions, our train evidently being the cause. Just before getting into Matjesfontein we looked out for and saw the grave of General Wauchope. Matjesfontein itself we admired, with its trim, well-kept station. We passed several trains after this bringing more of our troops to mobilise at Matjesfontein, where they expected to form one of the forces to go out to meet the rebels on their downward march. Tuows River, which is the beginning of the Karoo desert, is a railway depot, and here a sweet little woman came to the train with some fruit for us. The people of this community have a fund amongst themselves, and on certain days of the week they arrange in turn to cook suitable things for invalids, and they meet all ambulance trains as they pass through always with a meal ready for them. Our visitor told us that the eagerness and willingness of all to help was wonderful. She said that all the water they had was brought by the Government (in pipes) from the mountains, a distance of twenty miles away. Rain is so unusual an occurrence that when a few drops fell a short time ago her little girl of three was quite puzzled, and asked if the stars were crying!

### Arrival at the Camp.

We reached our camp, pitched at the foot of a range of hills in this Great Karoo plain at mid-day on Saturday, and were met by the medical staff and orderlies, who arrived here a fortnight ago. Our tents and huts are not all up yet, but already we cover about a quarter of a mile square. The building operations are under the supervision of two brothers, refugees from Johannesburg, who are giving their services, as also are many other of the workers in this little canvas town. The huts are in reality galvanized iron buildings lined with wood, there being a space between the two which admits air and keeps the place cool. The wards of 20 beds are all built in this manner, while there are as well many tents of different shapes and sizes set apart for patients. We are between 4,000 and 5,000 ft. above sea level. It is hot, but might be much worse. As this is one of the important watering stations we are fortunate in not being short of water, though, of course, it has to be carried some distance.

### Collapse of Tents.

To-day we have had some experience of what one sometimes has to put up with in camp life. During lunch the wind, which had been blowing in strong gusts all day, increased in violence. Some of us, anticipating a probable collapse of the tent, hurried out as soon as lunch was over; it collapsed at one end almost immediately afterwards, one of the poles pinning Miss Fisher to the table till she was released by some of the sisters rushing to the rescue. Fortunately, no damage was done. Excitement ran high when a large tent in which 20 of the sisters are sleeping, temporarily, also collapsed, some of the sisters inside almost losing their way in the descending billows of canvas. However, all emerged safely. It was a strange scene: a huge collapsed tent on the top of beds and baggage, a group of sisters, men dragging the canvas closely over beds and baggage to protect them from the heavy drops of rain now beginning to fall, while on the fringe of the crowd were the ever-familiar Kodaks snap-shotting the scene.

### A Plethora of Photographers.

We are quite resigned to being photographed at all times. On the last day on board we grouped ourselves, at the request of one of the passengers, and were taken by no less than six cameras at one time. The sisters who possessed cameras regretted having packed theirs, as it would have been quite worth while photographing our photographers. The moon is shining gloriously. Last night, before going to bed, several of us went for a stroll, coming face-to-face with a sentry. He called, "Halt! Who comes there!" Upon our replying, "A friend," he answered, "Pass, friends; all's well." I believe that after dusk this form is always gone through. It was strange to hear the stillness of the night broken by the cries of the sentries, one saying, "Ten o'cl ck; all's well," taken up by the next, "Number two; all's well," and so on from one to the other. Our beds have been apportioned, and we are all more than eager for work when our first batch of patients should arrive.

### To Nurses.

We invite contributions from any of our readers, and shall be glad to pay for "Notes on News from the Nursing World," or for articles describing nursing experiences, or dealing with any nursing question from an original point of view. The minimum payment for contributions is 5s., but we welcome interesting contributions of a column, or a page, in length. It may be added that notices of entertainments, presentations, and deaths are not paid for, but, of course, we are always glad to receive them. All rejected manuscripts are returned in due course, and all payments for manuscripts used are made as early as possible at the beginning of each quarter.

### Across the Seas.

### PRIVATE NURSING IN NATAL.

By a South African Nurse.

I HAD taken a room in a private house, a good half-hour's walk from town. The two windows opened out on to a verandah, in the garden beyond, hanging temptingly low on the branches of the trees, were numbers of oranges and lemons, looking in the sunshine like golden balls, the green leaves forming a velvet background. The doctors had been very kind and ready to help me all they could, and not a fortnight had passed before I was sent for to a case. The heat was intense, and I had just returned from the post office—the English mail having arrived that morning. On looking up after reading the dear home news, I saw a kaffir coming across the garden with a note for me, asking me to go off at once.

### My First Patient.

Scrambling my things into a bag, I went with the kaffir, who had a rickshaw waiting for me. We soon arrived at the house, a dark, damp place not far from the river. My patient had come from up country, and had taken the house furnished for a few months without having seen it. There was only my patient, her three children (the eldest just six), a kaffir woman to look after them, a man to do the housework, and myself in the house.

My patient had made all the necessary arrangements, and the woman had been shown the doctor's house, so that when I should want him she would know where to go.

### An Unpleasant Adventure.

A few nights after my arrival I wished the doctor called, I wrote a note to the doctor and a pass for the kaffir, as kaffirs are not allowed in the streets after 9 p.m. without one, and went to the room where the woman slept with the three children. To my consternation I found that she had gone, but I afterwards learned that it is a common occurrence for kaffir servants to run away from their situations. The kaffir man was my next resource. He slept in a little wooden house at the far end of the yard.

It was a terribly dark night, and it took me some time to find the door of his room; then I knocked and knocked, but it was quite a quarter of an hour before I managed to wake him. At last a tall figure wrapped in a blanket appeared in front of me, his two large eyes and rows of white teeth gleaming in the darkness. I could speak only a few words of kaffir, and the man understood about as much English, so quite a pantomime followed. At last, after saying about a dozen times, "missus very sick, fetch mutie man" (mutie is medicine in kaffir), he seemed to understand, took his pass and the note for the doctor, and departed. On returning to the house I found all three children screaming at the top of their voices; as there was no one else in the house I had to leave them and return to my patient. Two hours passed—they seemed more like twenty—the concert still proceeding in the children's room. Still no signs of the kaffir or the doctor. At last, just as daylight dawned, I heard footsteps approaching, and saw the kaffir coming up the path, followed by the doctor. We might have waited and waited, had not the doctor's boy quite by accident discovered our kaffir seated under a tree in the doctor's garden, waiting, as he said, "until the Boss should wake up." We had a lively time for the next hour; the heat was intense, and we had to work with windows and doors locked to keep out the children, who, finding their efforts were in vain to get into the room, kept up a constant knocking and screaming at the door. At last there was a lull, and upon opening the door we found the three seated on the floor in their night clothes, a large cup of coffee in one hand and a huge slice of cake in the other.

### Catering for Nurse.

We had many amusing scenes during the next few weeks, and the interviews I had with that kaffir man would do for *Punch*. He always bought the chickens alive, and kept them in the yard until they were wanted, and many a night on going to close the dining-room window I found our next day's dinner roosting on the window ledge. The following morning, whilst we were having breakfast, the kaffir would come into the yard, catch the fowl, and there, in front of the window, chop off its head, much to the amusement of the children and the hasty conclusion of my breakfast. During that month I had my first experience of the hot winds, when one has to keep doors and windows closed as the breeze is like a blast from a furnace. Private nursing is a totally different thing in South Africa to England. In Natal one often has to be cook, housekeeper, and nurse to the children, as well as attend on the patient. But on the whole I prefer the life, the people are so hospitable and kind, and when once one gets accustomed to their ways of living, and to having kaffir servants, upon whom one rarely can depend, the homesick feeling vanishes—at least, such is my experience.

### Minor Appointments.

City Hospital, Birmingham.—Miss Janet K. Mitchell has been appointed Night Superintendent. She was trained for three years at the Royal and County Infirmary, Perth, and for two years subsequently has had charge of the scarlet fever wards in the same institution.

Chalmers' Hospital, Edinburgh. — Miss Margaret Hamilton has been appointed Charge Nurse. She was trained at Dumfries and Galloway Royal Infirmary, and has since been night superintendent in the same institution, and head nurse in Kirkcaldy Cottage Hospital.

Beckett Hospital, Barnsley.—Miss Annie S. Wyatt has been appointed Charge Nurse. She was trained at the Royal Hospital, Portsmouth.

### Presentations.

Fiji Government Hospital.—An interesting presentation has been made by the medical officers of the Colonial Government staff in distant Fiji to the sister of the Government Hospital, Miss May Anderson. This lady received her training at the same institution, where she entered as a probationer in 1894 under the auspices of Miss Webster-Wedderburn, from the Nightingale Training Institution at St. Thomas's Hospital. Miss Anderson has risen by successive steps to the position of sister-in-charge, and has attained special distinction. Last year she underwent a trying time during an unhealthy season and an epidemic of tropical enteric fever among the ships of H.M. squadron at Samoa, and it was deemed advisable to grant her vacation leave to visit Australia and recruit her health. The medical officers with whom she had been associated availed themselves of the opportunity to present Miss Anderson with a complimentary address, engrossed and illuminated on parchment, which was accompanied by a return ticket to Australia by mail steamer, and a purse of sovereigns.

### Wants and Workers.

Cottage Home for Babies.—A certificated midwife wishes to start a home for babies in the country, and would be very glad if any district nurse or others could give her information of a suitable cottage in a healthy district. Three bedrooms would be sufficient, as it is intended only to start with a small number.

### Her Wedding Day.

By a Hospital Nurse.

Down a quiet London street a man and woman were walking. The woman was young and pretty, but her fair face was flushed, and her eyes red with crying. The man looked about forty; he was big and burly, with a good-tempered, good-looking face, which bore just now, however, an expression of worry and annoyance.

"I can't help it, Polly," he was saying, "it isn't my fault. You did it of your own free will. You saw how miserable I was, and said you would come and do for me, and I've promised over and over again that I'll marry you directly she dies."

The girl burst out crying again. "But, Joe," she said, "it's six months ago, and you told me then that she couldn't live a week, and my mother always brought us up respectable, and I don't know what she'd say if she knew your wife wasn't dead at all or us properly married, and she's always asking me where you go every Sunday afternoon that you can't go home with me and see her."

The man looked vexed. "Well," he said, "I think it's the least I can do to go every Sunday afternoon to ask how the poor old gal is. She's been a good wife to me for twenty years."

The girl whimpered again. "I believe you care more about her than me," she said; "and she may live ever so long, and our baby may be born before we can be properly married."

"Oh, Lord! Polly, how you do worry," the man replied. "What can I do? I can't kill her, can I?"

In a hospital ward a nurse was dressing a patient. For six long months the faded violet gown had been folded away, and now the poor woman who was to wear it was all tremulous with expectation. One arm hung almost helpless at her side, one leg dragged as she walked, the muscles of her face were not quite under control, her speech was slightly impeded. But her eyes were bright and intelligent, and her face flushed and eager.

"I don't think you're fit to go home to-day," the nurse was saying.

"Oh, I must, nurse," the woman replied. "You can't think how I want to get back home. My husband, he's such a man, he always wanted me to help him do everything. I can't think how he's got on so long without me. Fancy me being ill six months and not knowing anyone. And he's been to see me every Sunday all the time?"

"Yes," replied the nurse, "every Sunday. He'll soon be here now."

The woman trembled visibly. "Shall I tell you why I want to go home to-day, nurse?" she said. "It's because it's my wedding day. Twenty years ago to-day me and Joe was married, and he's been a good husband to me. We never had no children, and he's been like husband and child too. I waited on him hand and foot."

Suddenly the ward door opened, and her husband entered. His first glance was at his wife's empty bed, and a sudden pallor overspread his face. But the nurse smilingly indicated the chair by the fireside, where his wife, flushing and trembling like a girl, awaited his coming.

Joy leaped from his eyes, something in his throat seemed to choke him. Polly was quite forgotten. His own dear old girl, the wife of his youth, the faithful companion of twenty years, was given back to him from the grave. He kissed her almost reverently, then put his head on her shoulder and sobbed aloud. Poor soul, it was her one happy moment.

Her first words brought him back to realities.

"I'm so sorry I've been ill so long, Joe," she said, apologetically. "How ever have you got on without me? But I'm coming back with you to-day, darling."

The man started violently. Oh, what a fool he had been! If he had only guessed that his wife would recover he would have had nothing to do with Polly. For he was not all bad, only his rule of life had always been not what was right, but what was easiest, and he was very much under Polly's thumb now.

"Coming back with me now, are you?" he said, with forced cheerfulness. "That's right; that'll be like old times again. I'll just run and find a real comfortable cab for you." And he was gone.

But the real comfortable cab seemed hard to find, for the hands crept round the clock, the visiting hour was over, and the man did not return.

Nurse Milly looked anxiously at her patient. This was the first time she had been up, and her drooping attitude showed how utterly weary she was. At length she spoke.

"Mrs. Jenkins," she said, kindly but firmly, "you must go back to bed. Something has detained your husband, and it will be too late now for you to go home to-day."

The woman's drawn face was twitching, she only controlled herself by a strong effort. Can she have guessed the truth?

"Oh, nurse," she said; "and it is my wedding day!" And without another word she went back to bed.

It was Nurse Milly's evening out. Full of sympathy for her poor patient, and knowing the address of her home, she wended her way thither. The landlady opened the door.

"Can I see Mr. Jenkins?" she asked.

"No, you can't," said the landlady, irritably. "Such goings on I never saw in my life. After living in my two top rooms for years, this afternoon he comes in in a fine old state, a cart behind him, and his wife a-crying; and every blessed stick and stool have they moved away this Sunday afternoon, and where they've gone to I don't know and don't care."

"His wife crying?" exclaimed Milly. "What do you mean?"

"Just what I say," returned the woman. "His first wife's been dead six months. She 'ad a stroke sudden, and was took away to hospital. And that man, he couldn't do a hand's turn for himself. In about two days them two rooms was like a pigsty. I don't 'old with waiting on men so, I don't. My 'usband, 'e's as 'andy about the 'ouse as I is myself. Well, in about a fortnight he brings home a chit of a gal, and tells me as 'is wife died directly she got to 'ospital, an' 'e's married again. 'More shame for yer,' I says, 'an' a good wife like yours not cold in 'er grave.' But what he's up to runnin' off like this I don't know. 'E's paid 'is rent reg'lar."

Nurse Milly was met at the ward door on her return by the probationer, much excited.

"Oh, nurse!" she exclaimed, "Mrs. Jenkins has had another stroke, and Dr. Hammond says she won't get over this one. He says she got over-excited this afternoon. But she's worse than she was at first, and doesn't know any of us."

So the pitiful tale was never told.

### A Method of Cold-Water Treatment.

It may be interesting to record a case of typhoid in which the cold-water treatment was carried out in a novel and very successful manner. The patient, a young woman, with a temperature of 105·2, was placed, by the doctor's orders, on a cold-water bed until her temperature fell two degrees. This was done daily, and sometimes twice a-day, as long as the temperature rose to 103. This plan was carried out with a minimum of fatigue or discomfort to the patient, who said "it was far less horrid than the 'cold sheeting,'" which had been first tried.

### The Nursing of Dysentery.

By a Nurse from the Tropics.

II.

It is of the utmost importance for the nurse to take note of the dysenteric motions. The doctor will usually require one typical motion of the twenty-four hours to be kept for his inspection. Wide variations exist in the motions of different cases of dysentery. Throughout the entire case there may be nothing more characteristic than the orthodox mucus and blood. Occasionally the whole motion may look like a bundle of vermicelli tinged pink with blood, the mucus composing the bundle being very tough and stringy. At other times the motion appears as a collection of marbles, coated with mucus or pus. This marbling of the motions is very characteristic, and differs widely from the well-known condition known as the passage of scybala. The nurse must distinguish between mucus and pus—the latter points to the presence of abscesses, as distinct from ulcers, in the intestines. In most cases of dysentery the whole digestive system appears to be out of order. The food may be passed almost unaltered, and the patient suffer from severe dyspepsia.

Motions should be disinfected carefully and throughout. It is not proven that dysentery can be communicated from one person to another. But it is not certain that it may not be. It is a germ disease, and some forms are parasitical, so that sedulous disinfection is desirable.

### The Cure of the Disease.

Dysentery can only be cured by the subsidence of the congestions and inflammation of the simpler cases, and by the healing of the ulcers and abscesses of the more severe type. Owing to peristalsis, and the constant passage of food over the inflamed intestinal surfaces, the ulcers may take long in the healing. Absolute rest and a suitable diet are two chief nursing points. No diet which excites peristaltic action of the bowels or acts as a stimulant to the mucous membranes is permissible. Bland, soft, easily-digested foods, unspiced and unflavoured, are desirable.

### The Question of Foods.

The next point is to choose foods which leave little fæcal residue. The natives of tropical countries aver that milk is a deadly food for dysenterics. Few natives with dysentery will touch a drop of milk.

Native physicians in India mostly declare that a high death percentage follows a milk diet in this disease. The argument rests on the fact that milk, being so liable to fermentation and decomposition—especially in a tropical country—forms a ready nidus for bacteria and intestinal germs generally.

The writer, with a somewhat wide experience of dysentery, confirms the native belief that milk is very badly borne by dysenteric patients. If given it should be highly diluted with barley water. Generally speaking the patient will do better on whey, chicken, or mutton broth. Rice or barley should always be cooked in these broths and carefully strained out. Valentine's meat juice, patent malted milk (not fresh malted milk), are both admirable. Beef-tea extracts tend to excite diarrhœa.

White of egg, either whipped into broth or shaken up in a bottle of broth or barley-water (like the egg-water for diarrhœa), is excellent. Condensed milk or the prepared peptonised cocoa and milk are both good. Dysentery being often associated with a scorbutic state of the blood, a dessert to a table-spoonful of carefully-strained lemon or orange juice may be given daily. Brandy will invariably be prescribed for the patient. The water he drinks is an important point. If distilled water, or a reliable mineral water, cannot be procured the nurse must superintend the filtering and boiling of the water given to the patient. So often the disease is caused by the water supply, and to continue the polluted drink during the illness is to seriously handicap recovery. Condensed milk flavoured with cinnamon is good. The latter is believed to have a specific action in dysentery. All food should be given tepid. Hot and very cold liquids are too stimulating to the intestines.

In the early stages ipecacuanha is usually prescribed. In severe cases not yielding readily to drugs the "Maclean treatment"—that is a large bolus of ipecacuanha—is given in order to bring the patient completely under the influence of the drug.

### What the Nurse Can Do.

The nurse can do much to render this treatment efficacious. Many patients are so susceptible to ipecacuanha that violent vomiting frequently follows the bolus. Extreme nausea is always caused by it, and if this go on to vomiting the value of the treatment is spoiled by the rejection of the drug. The doctor generally gives special directions with regard to this. Usually a three-hour fast will be enjoined before the ipecacuanha is given. Dysentery patients are always ready to fast. They have little appetite or desire for food. About twenty drops of laudanum in half an ounce of water twenty minutes before administering the Maclean bolus is an orthodox practice; the laudanum being followed in ten minutes by the application to the epigastrium of a mustard poultice. This should be kept on as long after the ipecacuanha has been taken as the patient can endure. It tends to prevent vomiting—nothing counteracts the extreme nausea following this treatment. The patient needs absolute quiet and rest in a darkened room whilst under ipecacuanha influence, neither speaking nor moving. He is almost afraid to breathe for the extreme desire to vomit. Ice is not allowed in dysentery, but a little may be swallowed to allay the ipecacuanha nausea. These boluses may be continued for several days if the first two do not cut short the active symptoms of the disease. In the ipecacuanha stage of the disease food must be given cautiously and in small quantities. Practices differ with differing cases. Very commonly a dose of castor oil and laudanum will be given the night before the ipecacuanha "dosing" begins. Sometimes calomel takes the place of the oil. Many doctors, however, prefer the use of enemata. Barley water or linseed tea injections are ordered for washing out the bowel. A pint of barley-water and quarter drachm of boracic acid is a usual daily enema to prevent fermentation and to keep the bowel aseptic. If much diarrhœa be present and the patient thus exhausted and kept awake by persistent night motions it is often necessary to give a small enema of half ounce barley-water, with 20 drops laudanum and quarter drachm bismuth powder. This enema to be retained all night, and followed in the morning by the above-mentioned boracic and barley-water injection. Irrigation of the bowels is often very valuable. The boracic acid appears to relieve tenesmus, for which also cocaine suppositories may be ordered. Injections need to be given slowly, and the tube well oiled and tenderly introduced, owing to the ulcerative and sore condition of anus and rectum.

### The Danger of Relapse.

As the patient gets better constipation is sometimes a troublesome feature. The use of aperient medicines is very likely to bring back all the dysenteric symptoms. Small doses of castor oil or glycerine suppositories are the safest remedies, since stronger measures are apt to reproduce the dysentery. Constipation can hardly be overcome in the diet, since stewed fruits, brown bread, &c., are not permissible for many months after an attack of dysentery.

Warm clothing must be worn and the patient carefully guarded against chills. After dysentery the least intestinal chill is apt to be followed by abdominal pain and severe diarrhœa. Cold bathing is not permissible to those who have ever had an acute attack of dysentery. The seaside has a specially beneficial effect on dysenterics, but sea bathing must be absolutely interdicted. A patient may be apparently well for many months, and he then relapses suddenly into an acute attack. Intestinal thickening and the contraction of the bowel caused in the healing of the ulcers often follows as well as a general wasting owing to the unhealthy state of the alimentary canal and a consequent non-absorption of nourishment.

The Necessity of Strict Dieting.

So long as the nurse has charge of the case, she must impress on her patient the absolute necessity for strict dieting. The least relaxation of caution in this direction is apt to set up congestion and diarrhœa. Red meat will need to be taken with caution during convalescence. White soups, poultry, eggs, fish, and milk puddings are the foods on which the dysenteric patient does best, maccaroni well boiled is believed in the tropics to be a most valuable food for convalescents from this disease. Very little milk should be taken in fluid form. Made into puddings and long cooked it is admirable. The nurse must daily examine the motions of the convalescent dysenteric. Sometimes for months in a relapsing case these will be most unhealthy, offensive, and containing sloughs and mucus. The least appearance of blood in the stools must be reported to the doctor. In point of fact the dysenteric patient needs as careful nursing as though he were a typhoid. But the period of possible relapse lasts much longer in the former than in the latter illness. Dysenterics are extremely interesting cases and by no means monotonous. The course of the disease differs so widely in different patients.

### The English Hospital, Haifa.

By a Special Correspondent.

### THE PATIENTS.

Our small English hospital at Haifa, though professedly for the Jews, is cosmopolitan. Most of our patients are Arabs, though we have a few negroes, Turks, and Jews. We have also had Germans, Italians, and even Englishmen. We never refuse anybody unless we cannot possibly take them in. Some of the patients are very dear people, particularly the Moslems. Many of the women are good-looking, and the men handsome. They are all full of the prettiest of quaint sayings, which they shower down upon you whenever possible. They kiss your hand whenever they can, and hold it up to their foreheads, which is a token of great esteem. I have even had the folds of my dress caught and kissed by a man who was very ill indeed. The Arabs are an impulsive, warm-hearted, indolent, fanatical race. They are also very bright and intelligent. It is often rather difficult to maintain order in the wards; but a little kindness, firmness, and tact go a great way. On the whole, they are very obedient and well-behaved, especially when one realises how severe our restrictions must seem to these untamed sons and daughters of the desert, with all their nomadic habits. Sometimes we have great difficulty to keep the men in bed, and they will smoke.

### The Passion for a Smoke.

When I first came here I was on night duty. One night an old Jew, who was nearly dying, tried to make me understand what it was he wanted. I suggested every possible thing I could think of, to each of which he shook his head and said, "La, la" ("no, no"). At last he pointed under his bed, but the only thing I could find was a dirty book of cigarette papers. His wan face lit up as I, in utter astonishment, held it to him. He seized the wretched little book and, ill as he was, unfolded an old pocket-handkerchief he had hidden in bed. From one corner he took out some tobacco, and from another a box of matches. Then he deliberately rolled himself a cigarette, struck a light, and began to smoke. His joy was so great that I had not the heart to stop him, though I limited him to one cigarette, and stayed by his side until it was quite finished, when he meekly submitted to having his tobacco and matches confiscated for the remainder of the night. In the daytime all the men patients are allowed to smoke, and each has an ash-tray on his locker. Apart from the question of the advisability of permitting such a thing, it seems curious that sick people should care about smoking.

### The Language Difficulty.

One of the many great difficulties which beset a new nurse is not knowing the language of the people. This is especially felt at night, when the interpeter is asleep. For the first few weeks after my arrival it went to my heart not to know what the patients said. Of course, one soon picks up words, and I am now having Arabic lessons. But at first not knowing the language is like a wall between you and the people. My own experience was rather a bitter one. Here was I, a ne arrival, not knowing one word of the language, nor th patients, nor the ins and outs of the hospital, on night duty single handed! Many a time as I started to go on m rounds I have drawn back for a minute to listen to som curious noise, and often half-expected to see a dusky figure moving downstairs or peeping in at one of the windows! But nobody ever appeared, and I soon lost my fears. The men patients, who knew I was a fresh comer, were evidently amused at me, and one large Bedouin, of whom I really was at first afraid, used to sit bolt upright in bed whenever I went near him, and fix his enormous dark eyes upon me. He always said. "Ma salame" as I went out of the ward. Wishing to keep on good terms with him, one night I returned his salutation, whereupon the whole ward laughed! I learnt afterwards that "Ma salame" means "Go in peace," and is, of course, only said to a person leaving a room, or setting out for a walk or a journey.

### Our "Cases."

The patients are very grateful, and have the utmost respect for anyone who can alleviate their pain. They think one must be gifted with almost miraculous power, and pay honour accordingly. They do not like you to be too gentle, though, because, poor souls, they imagine that to be cured they must be hurt—made to feel bodily pain. Their own methods of treating sickness are absolutely barbarous. For instance, for many illnesses they burn themselves with hot irons, and often cut deep flesh wounds with knives to "bleed" themselves. One man who took his child, suffering from whooping-cough, to the doctor, said, "I've burned his chest and I've even burned his back, and still he has the cough!" Many of our patients have ugly scars from burns of this kind.

### The Dread of Consumption.

All natives have a great horror of consumption. A consumptive patient is avoided almost as much as a leper. He has everything for his use separate, from the food, linen, &c., of the other members of his household. When he dies the people pull down his house. Small-pox they do not dread half so much, nor do they take precautions to avoid contagion of this loathsome disease. The chief cases brought to the hospital are malaria, typhoid, nephritis, dropsy, and heart complaints. We do not have many accidents nor a great number of surgical cases. Most of our operations are minor.

### Echoes from the Outside World.

AN OPEN LETTER TO A HOSPITAL NURSE.

SURELY divinity doth hedge an Heir Apparent as well as a King, or four charges fired at close quarters from a six-chambered revolver would have resulted in something worse at Brussels last week than a scare. How easily the shots might have done serious injury has been proved by the discovery of one bullet at the back of the sofa where Miss Knollys and Admiral Stephenson were sitting, which must have passed exceedingly close, not only to the Prince of Wales, but to the lady and gentleman-in-waiting. Young Sipido, whose parents express great surprise that he could have been capable of such a crime, belongs to the Social First Advance Guard of St. Gilles, which consists of 400 young Socialists, all under twenty years of age. The Prince of Wales has, it is stated, expressed a hope that the incident should be treated as the outcome of some youthful wrong-headedness, but that is probably more because he does not wish to create unpleasant relations with a nation which has hitherto shown him much courtesy, rather than because he thinks an attempt at assassination, whether the consequence of a bet of five francs, as Sipido maintains, or of Socialistic antipathy to Royalty, should go unpunished. That such an association should number so many evilly-disposed youths bodes ill for Belgium, but meantime it is pleasant to reflect that not only are the Prince and Princess uninjured, but that so profound is the universal feeling of satisfaction at his escape that the Prince has already received at Copenhagen nearly two thousand telegrams. It was feared by those who know little about the Queen that this deplorable incident would interfere with her showing herself in public in Ireland. But courage has always been one of Her Majesty's characteristics, and she has not manifested the slightest disposition to curtail her daily drives around Dublin. There is no doubt that, all being well, she will visit Belfast on her return journey.

---

AGAIN, we have comparatively no news from the war. It is not known if this is because Lord Roberts has succeeded in keeping secret any plans he may have formed, or because the wires have been cut. The latter event is anticipated, and will cause no serious alarm. The disaster at Reddersburg naturally saddened us all, not only because of the men we lost, but because it shows how extremely difficult it is to be even with the Boers except in big movements, or to persuade British officers to be cautious as well as courageous. It is reported that the Boers are preparing a flanking movement to get round the British position at Wepener, and to do so are ready to invade Basutoland. I hope they may, for the Basutos are ready to give them a warm reception. The information given to the Mafeking garrison from Lord Roberts that arrangements should be made to hold out till May 24th, seems to be well founded; and the poor half-starving defenders of the little garrison, after six months of imprisonment, appear to have received the tidings with curious resignation. But its relief is the event which, of all others, would be received with joy wherever the English language is spoken.

---

NOW that the mails are running freely, every letter which comes from Natal is full of accounts of the experiences at Ladysmith. People who bore the brunt of those dreadful months have escaped as soon as possible from their late prison, and gone to recruit themselves at the seaside or at health resorts. My correspondent says: "But in many instances it will be months, if not longer, before health is restored. Not only has the want of good food seriously enfeebled many constitutions, but the nervous system seems to have been completely shattered in some instances. All the horrors of the siege have so affected one officer that the slightest noise, even the dropping of a spoon, will cause him to run and hide under the table; and even from the boys' faces all the youth seems to have vanished. One boy of 19 with whom I had a long talk looked 30, and talked like 60. The sad eyes and the hollow voice told their dreary tale all too well, and it was only quite towards the end of our chat that a few boyish phrases crept out. He said he had felt the hunger keenly. In the last ten days the 1¼ biscuit was served out in the evening, and few had sufficient self-control to keep any of it to go with the tablespoonful of horse mince which was served at midday. During the rest of the 24 hours there was nothing. Those men who were able to secure more horseflesh, and ate it in any larger quantities, were avoided by their companions, as their breath smelt so badly."

---

"To some of the besieged the want of letters and personal news caused even more distress than the want of food. Although they felt sure their relatives had written, never a line came, because though it was comparatively easy to get 'boys' to leave Ladysmith, it was extremely difficult to get any who were willing and able to bring news into the town. Many attempts were made. One native started with 300 letters, but threw them away on the road, and, with strange irony, it was the relieving column who ultimately brought them in. On another occasion six runners were sent from Estcourt with forty tins of milk, and were paid £15 apiece for the job. Two got frightened as soon as they reached the Boer lines, and dropped their precious freight; two more were made prisoners by the Boers, but the last two succeeded in hiding their burden as soon as they saw they were likely to be searched, and after they were released again—nothing having been found upon them—they went and recovered not only their own hidden tins, but the eighty abandoned by the first two men. Ultimately they arrived safely in Ladysmith carrying 160 tins between them! A little boy, who had been worrying much about his father's health, was fortunate enough to secure one for 5s., and carried it off in triumph, refusing to part with his treasure, although he was offered £1 1s. for it long before he got up the street. He always avers that that tin of milk saved his father from a serious illness."

---

THE case of Madame Zuleika, who was charged at Marlborough Street Police Court last week with pretending or professing to tell fortunes, has excited a great deal of interest, because so many of us love a spice of mystery. Those who are too full of common-sense to believe in witchcraft themselves frequently practise the "art" at a "palmistry" or "gipsy" tent for the benefit of the funds of a bazaar; and if the decision that "the defendant had brought herself within the meaning of the Act," with a penalty of £25 and costs to pay, be upheld, some of us will have to mend the error of our ways. Notice of appeal has been given, so that the matter is not yet settled. I remember once going with a friend to consult a lady of the "Madame Zuleika" type, who also had her home in Bond Street. She was one of the prettiest girls I ever saw, dressed in lovely Eastern robes, her alcove redolent with sweet scents, radiant with flowers, and glowing with gorgeous drapery, whilst her manners were very bewitching. My friend made me go in first, because she was too timid, but I am afraid I was too much of an unbeliever to be a good subject, for she could make little of me, and only told me to beware a date in May, which I straightway forgot next day. But the lady I went with emerged from the sanctum in a high state of indignation, for the soothsayer had told her she was either living apart from her husband, or had been divorced. As they had only been married a couple of years, and were most devoted, the guess was very wide of the mark. I reflected, as I quitted the salon, that that particular "wise woman" had more beauty than wisdom.

### Everybody's Opinion.

[Correspondence on all subjects is invited, but we cannot in any way be responsible for the opinions expressed by our correspondents. No communication can be entertained if the name and address of the correspondent is not given, as a guarantee of good faith but not necessarily for publication, or unless one side of the paper only is written on.]

### THE RECORDING CLOCK.

"UNION INFIRMARY" writes: A well-wisher of hospitals has my sympathy; I was very glad to see her letter, more especially on the grounds of the principle of the thing. I also am far too fond of my work to need such reminders, and I think they tend to lower our standard as nurses rather than raise it. The sooner "public opinion" changes its opinion of nurses, more particularly workhouse nurses, the better. The noise, too, of our correspondent's clock must be very annoying to the patients, though the one I have to use when on night duty is quite silent, and no noise is incurred in using. The annoying part is the apparent want of confidence displayed by those who enforce its use.

### DRESS AT A NURSES' DANCE.

"S. E. B." writes: It would be difficult indeed to express the disgust and chagrin with which I have read one "Note on News from the Nursing World." I refer to the extracts of a report given in a local paper concerning the annual dance at the Wandsworth and Clapham Infirmary. What a miserable sense of degradation the reading gives rise to in one! It falls so deplorably short, not of one's ideals, but one's ordinary everyday standard of work—nurses and nursing. In charity one would fain believe the report to have been made by some spiteful and little informed "outsider." One blushes in solitude for those "belles"—names given, too! Was the report given by one of the "few gentlemen"? Save the mark! And I have been an advocate of dancing! Of a truth the cure is worse than the disease.

### "THE STAGE NURSE."

"NURSE FROM THE COUNTRY" writes: I am a trained nurse from the country come to London to see the sights, and amongst other things I went to see the play called "Nurse," at the Globe Theatre. I believe they term it a farce. Well, a farce it certainly is, in the true sense of the word. Curiosity led me there. I wondered how they could make the life of a nurse the subject of buffoonery. As a rule, she spends her day amidst sickness, suffering, and death, and it puzzled me to know what attraction these sad conditions could have for the audience of a comedy theatre. The whole thing is a misrepresentation. Unfortunately, however, one-half of the audience will not know that, having probably never come in contact with the true nurse, and it is quite evident that the actress herself knows next to nothing about the character which she has utterly failed to impersonate. Her dress is made with a train, round her waist she sticks pins to prevent her patient from putting his arm there, and yet she sits on the lounge where he is reclining. After placing the thermometer in his mouth, she leaves the room until it is time to take it out. She shrieks and dances about in quite a hysterical manner when anything unexpected takes place. She constantly interrupts the doctor while he is giving his directions. Altogether, I am afraid that most of those present would go away with the impression that a trained nurse is, as a rule, a fast and objectionable young woman.

### PROMOTION IN INFIRMARIES.

"PROBATIONER NORAH" writes: I was glad to see a letter on the subject of promotion in infirmaries, and would be glad if you could spare me a little space in your valuable paper for a few words on the same subject. I quite agree with what a "Nurses' Friend" says, and I certainly sympathise with the staff of the infirmary mentioned. I myself know of a certain hospital (the name of which I will not mention, for if this letter is printed and is seen by the committee or matron the cap will fit without any explanation) where only last week a night sister was advertised for. This vacancy, and also another, that of assistant matron, have both been vacant on two occasions within the last eighteen months, and each time a stranger has been sought to fill these posts, while in the hospital nurses and sisters have been fully qualified to fill them. Moreover, several of them applied, and were politely told that a stranger was to be appointed. Now I think this very hard and most unfair to all the staff in the hospital. I do not know who is the cause of such injustice being done, but I do know that if the staff of any hospital fail to uphold each other no one else will. It is certainly most discouraging to nurses to feel that at the end of their year's training the matron and doctors are willing to give them splendid testimonials, but that when it comes to a little promotion in their own ranks a stranger is selected. Certainly, some people must be fond of strange faces in their midst. Personally, I have not suffered any such injustice myself, as I am only a "pro.," but I have friends who are victims of it, and so I take it up on their account, and hope that other fellow-workers will do the same now that the subject has been started.

### THE NEUROTIC NUISANCE.

"NURSE E." writes: Can nothing be done to abate the neurotic nuisance which is spreading and increasing throughout the land? Do people realise the number of homes (and I might add of husbands also, for it is generally the rich, childless wife who is the guilty one) that are darkened and mined by the defection of her who should be "the angel of the house"? She lives upstairs in darkened rooms, lying in state in a beautiful tea-gown, surrounded by flowers, and concentrates her whole attention on her inward parts; the most ordinary functions of the body are to her pathological. Her only literature consists of her bundles of prescriptions, which are constantly being perused, and perhaps her Bible, for these people have generally a sickly veneer of religion, and have their clergymen to see them. She is to be kept from all worries—in fact, from everything which does not turn on herself. She spends, or makes her husband spend, hundreds yearly on doctors and chemists, she runs one imaginary disease after another, her condition becoming more and more hopeless. All the finer instincts disappear, the animal part comes out in her; perhaps only a nurse knows how degraded she can become. She gets wholly absorbed in her own body, her very soul seems dead, till at length from a moral wreck she becomes a mental one, and the end begins. A case of long standing seems hopeless, temporary improvement being generally followed by relapse. But might she not be reclaimed in the early stages if she could only be made to realise that such self-indulgence is shameful and degrading? Neurotics would not like to be classed with inebriates, but surely there is no great difference between their failing and drunkenness or any other form of self-indulgence. Most of them hug their nerves, and seem to think it rather a laudable condition than otherwise. They are so highly organised that they are vastly superior to their fellow mortals. "My doctors all say I am so highly strung," one often hears them say, with a would-be pathetic smile; "what is only a little pain to you is agony to me." No doubt they do suffer more than ordinary people, but is that any reason why they should be endowed with less moral stamina than others—any reason why they should be encouraged to lead a life of utter selfishness, as so many of them are? Surely something might be done in the early days to prevent them drifting into actual disease and death (I am not referring to those cases of nervous exhaustion caused by great mental strain). If these neurotics could only be convinced that their state is one to be deeply ashamed of, that it actually amounts to the criminal, it seems that they might be induced to make more fight against it.

### For Reading to the Sick.

Now is Christ risen from the dead, and become the first-fruits of them that slept.—1. *Cor.* xv. 20.

Alleluia ! Alleluia !
Hearts to Heav'n and voices raise ;
Sing to God a hymn of gladness,
Sing to God a hymn of praise ;
He, Who on the Cross a Victim
For the world's salvation bled,
Jesus Christ, the King of glory,
Now is risen from the dead.

Christ is risen, Christ the first-fruits
Of the holy harvest field,
Which will all its full abundance
At His second coming yield ;
Then the golden ears of harvest
Will their heads before Him wave,
Ripen'd by His glorious sunshine,
From the furrows of the grave.

Christ is risen, we are risen ;
Shed upon us heavenly grace,
Rain, and dew, and gleams of glory
From the brightness of Thy Face ;
That we, with our hearts in Heav'n,
Here on earth may fruitful be,
And by Angel-hands be gather'd,
And be ever, Lord, with Thee.
Alleluia ! Alleluia ! —*C. Wordsworth.*

**Reading.**

Why was He buried ? In His grave He paused until the Easter morning. Hence the grave is the waiting-place for the great awakening. That great awakening shall surely come. Such is the Christian's hope. I had rather say because he walks by faith,' the Christian's certainty. . . . "Them that sleep in Jesus will God bring with him. . . ." We have the revelation in the quiet form on Good Friday night, the risen Jesus on Easter Day. As certainly as sleep implies awakening, so since Jesus was buried and rose again, the grave means resurrection from the dead ; means, in fact, that here we work, and there we wait ; wait for the great awakening. This is the solemnity of the mystery of the grave. . . . The sorrows of life, its partings, its disappointments, its defeats, become bracing and blessed when the eye is fixed on Him "who liveth and was dead," for so they can be borne in the strength of other memories as real, and more lasting than they. These are voices from another world, these are whispers from a better land, these are consolations when we think of others, invigorating thoughts for ourselves.

Sleep after weariness,
Rest after labour,
Peace after storm ;

To be "with Christ," the Ideal, the sympathising sufferer, the Saviour, the Friend ; these truths soften and illuminate the solemn mystery of the grave.—*Knox Little.*

Out in the rain a world is growing green,
On half the trees quick buds are seen
Where glued-up buds have been.
Out in the rain God's Acre stretches green,
Its harvest quick tho' still unseen :
For there the Life hath been.

If Christ hath died His brethren well may die,
Sing in the gate of death, lay by
This life without a sigh :
For Christ hath died and good it is to die ;
To sleep when so He lays us by,
Then wake without a sigh.

Yea Christ hath died, Christ is risen again,
Wherefore both life and death grow plain
To us who wax and wane :
For Christ who rose shall die no more again ;
Amen : Till He makes all things plain
Let us both wax and wane.
—*Christina Rossetti.*

### Notes and Queries.

THE Editor is always willing to answer in this column, without any fee, all reasonable questions, as soon as possible.
But the following rules must be carefully observed :—

1. Every communication must be accompanied by the name and address of the writer.
2. The question must always bear upon nursing, directly or indirectly.

If an answer is required by letter a fee of half-a-crown must be enclosed with the note containing the inquiry.

*Contracting Out.*

(20) In my opinion Poor Law Boards who deduct from salaries acknowledge liability for pension, whether the nurse contracted out or not. Please tell me, in your answers to correspondents, what you think on that point. If a Board, after deducting, refused a pension on the ground that the nurse had contracted out, she could recover amount deducted with interest. Don't you think so ? Will you also kindly tell me what pension a nurse, now aged 35, salary £45, deductions made, would be entitled to at the age of 50 ?—*H.*

"H." had better read the Act carefully. The question he asks is a purely legal one, involving a consideration of the exact wording of the Act. What is clear is that if a nurse in the service of the Guardians at the time the Act was passed contracted out in accordance with its provisions the Guardians could not compel her to contribute. "H.'s" difficulty probably arises from the fact that the nurse he is thinking of has left the service, and then on re-entering regards her contracting out as still operative. As to the question of the amount of pension to which a nurse would be entitled to at the age of 50, the answer is—none. Again we say, read the Act. Mere age does not entitle to pension until the nurse is 65 years old, and still at work. The nurse must have become incapable of performing her work, or she must have been 40 years in the service if she is to obtain a pension under the age of 65.

*Responsibility in Disinfecting.*

(21) Will you be so kind as to answer the following questions for me ? Supposing a nurse who is attached to a private nursing institution be sent by the principal of it to a case after having been nursing scarlet fever, and having no time allowed for quarantine, can she refuse to go ; and, in the event of her going and the deception being found out, would she, or the one who sent her, be liable to punishment ? Thanking you in anticipation for replying to this.—*Shamrock.*

Of course she can refuse to go. Whether such refusal would in the eyes of the law justify the institute in discharging her would be for the decision of a county court judge, according to the circumstances of each case. We do not, however, think the institute would risk the exposure involved in trying such a case, but would probably adopt other measures. The Public Health Act does not seem to take cognisance of the possibility of a *person* carrying infection, but it does make it unlawful to give, lend, sell, transmit, or *expose*, without previous disinfection, any bedding, *clothing*, rags, or *other things* which have been *exposed to infection* from a person suffering from a "dangerous infectious disorder," and for a nurse to go to a fresh case in the clothes which she had worn at an infectious case would clearly be an illegal act which no one could be compelled to do. Moreover, if she allowed herself to be persuaded to do it she would herself be responsible. We doubt, however, whether, according to the strict letter of the law, any period of quarantine could be enforced, unless evidence were to be adduced showing that the proper disinfection of some of the "other things" which the nurse must take with her, such, for example, as the nurses' hair, could not be effected without the lapse of a certain space of time.

*Maid Attendant.*

(22) "E. G." would be grateful if the Editor would inform her what is required of anyone taking the position of nurse-attendant or maid-attendant, as she often feels tempted to answer appointments under those headings, but that she does not quite understand what qualifications are needed, or what her duties would be.

Both the qualifications required and the duties to be performed vary with the exigencies of each case ; but a certain amount of nursing knowledge and a love of such work would be desirable.

*Nursing in India.*

(23) Will you kindly give me the address of the Up-Country Nursing Association, or, failing that, how and where I could obtain nursing employment in India ?—*L. W.*

The hon. secretary to the Up-Country Nursing Association is Major-General J. Bonus, The Cedars, Strawberry Hill. Full particulars of the Indian Army Nursing Association were given in our last number.

*Soldiers and Sailors' Families' Association.*

(24) Could you tell me the address of the Soldiers and Sailors' Families' Association ?—*Nurse G. B.*

The address is 23, Queen Anne's Gate, Westminster.

*Nurses' Home.*

(25) Could you tell me to whom I must apply to register a nurses' home ?—*E. W.*

There is no registry for nurses' homes.

*Standard Books of Reference.*

"The Nursing Profession : How and Where to Train." 2s. net.
"The Nurses' Dictionary of Medical Terms." 2s. 6d. net.
"Burdett's Series of Nursing Text-Books." 1s. each.
"A Handbook for Nurses." (Illustrated.) 5s.
"Nursing : Its Theory and Practice." New Edition. 3s. 6d.
"Helps in Sickness and to Health." Fifteenth Thousand. 5s.

All these are published by THE SCIENTIFIC PRESS, Ltd., and may be obtained through any bookseller or direct from the publishers, 28 & 29, Southampton Street, London, W.C.

## Figures and Tables

**Fig. 5. f1:**